# Synthesis of acyl oleanolic acid-uracil conjugates and their anti-tumor activity

**DOI:** 10.1186/s13065-016-0217-5

**Published:** 2016-11-21

**Authors:** Wei-bin Mo, Chun-hua Su, Jia-yan Huang, Jun Liu, Zhen-feng Chen, Ke-guang Cheng

**Affiliations:** 1 State Key Laboratory for the Chemistry and Molecular Engineering of Medicinal Resources, Guangxi Normal University, Guilin, 541004 People’s Republic of China; 2 School of Chemistry and Pharmacy, Guangxi Normal University, Guilin, 541004 People’s Republic of China; 3 Biochemistry and Pharmacology of Sport School, Guangxi Normal University, Guilin, 541004 People’s Republic of China; 4 Jiangsu Key Laboratory of Drug Screening, China Pharmaceutical University, 24 Tongjia Xiang, Nanjing, 210009 People’s Republic of China

**Keywords:** Acyl oleanolic acid, Uracil, Anti-tumor activity, Cytotoxicity, Apoptosis

## Abstract

**Background:**

Oleanolic acid, which can be isolated from many foods and medicinal plants, has been reported to possess diverse biological activities. It has been found that the acylation of the hydroxyl groups of the A-ring in the triterpene skeleton of oleanolic acid could be favorable for biological activities. The pyrimidinyl group has been constructed in many new compounds in various anti-tumor studies.

**Results:**

Five acyl oleanolic acid-uracil conjugates were synthesized. Most of the IC_50_ values of these conjugates were lower than 10.0 μM, and some of them were even under 0.1 μM. Cytotoxicity selectivity detection revealed that conjugate **4c** exhibited low cytotoxicity towards the normal human liver cell line HL-7702. Further studies revealed that **4c** clearly possessed apoptosis inducing effects, could arrest the Hep-G2 cell line in the G1 phase, induce late-stage apoptosis, and activate effector caspase-3/9 to trigger apoptosis.

**Conclusions:**

Conjugates of five different acyl OA derivatives with uracil were synthesized and identified as possessing high selectivity toward tumor cell lines. These conjugates could induce apoptosis in Hep-G2 cells by triggering caspase-3/9 activity.

**Graphical abstract:**
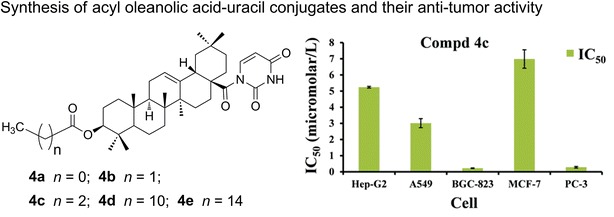
Five acyl oleanolic aicd-uracil conjugates were synthesized. These conjugates exhibited selective cytotoxicity toward tumor cells achieved via inducing apoptosis by activation of caspase-3/9.

**Electronic supplementary material:**

The online version of this article (doi:10.1186/s13065-016-0217-5) contains supplementary material, which is available to authorized users.

## Background

**Fig. 1:**
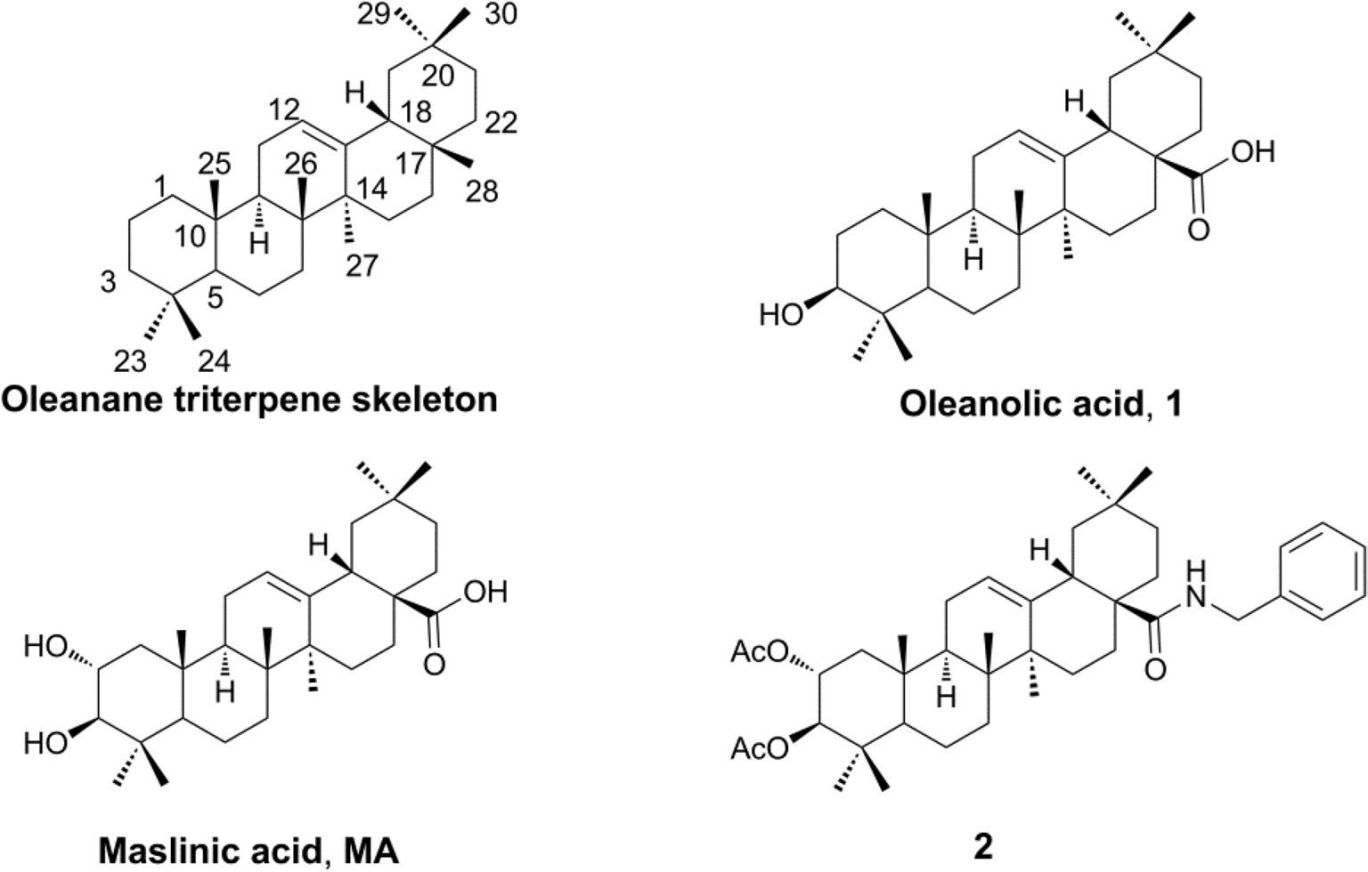
Chemical structures of oleanane triterpene skeleton, oleanolic acid, maslinic acid and **2**

Pentacyclic triterpenes, which are ubiquitous in the plant kingdom, have important ecological and agronomic functions, and contribute greatly to pest and disease resistance and to food quality in crop plants [1]. They are also applied in a variety of commercial uses in the food, cosmetic and pharmaceutical fields. For example, pentacyclic triterpene imberbic acid, isolated from *Combretum imberbe* (Engl. and Diels), has been found to have particularly potent activity against *Mycobacterium fortuitum* and *Staphylococcus aureus* [2]. Other pentacyclic triterpenes have been reported to possess antioxidant, antiproliferative, and pro-apoptotic capacities on MCF-7 human breast cancer cells [3]. They were also reported as a new class of glycogen phosphorylase inhibitors [4] and further proved to be multi-target therapeutic agents for the prevention and treatment of metabolic and vascular diseases [5]. Oleanolic acid (3β-hydroxyolean-12-en-28-oic acid, OA, **1**, Fig. 1), which belongs to the family of oleanane pentacyclic triterpenes, has been isolated from more than 1620 plant species, including many food and medicinal plants [6]. It is among the major effective components of some well-known traditional chinese medicines (TCM) such as Rehmannia Six Formula (Liu Wei Di Huang Wan), which is one of the most commonly used Chinese herb formulas in the world. It has been used as a nonprescription antihepatitis drug for almost 35 years in China [7]. Oleanolic acid and its derivatives have recently attracted much attention due to their diverse biological activities [8]. For instance, oleanolic acid and its derivatives were reported to be inhibitors of protein tyrosine phosphatase 1B with cellular activities [9] and osteoclast formation [10, 11]. These compounds were also focused on cytotoxicity evaluation [12]. Furthermore, some synthetic oleanane triterpenoids (CDDO, CDDO-Me and CDDO-Im) have demonstrated potent antiangiogenic and antitumor activities in rodent cancer models [13, 14]. Other biological activities of oleanolic acid and its derivatives, including antiproliferative activity in solid tumor cells [15], inhibition of α-glucosidase [16], and others [6, 8], were also revealed.

The importance of C-3 in the oleanolic acid skeleton was elucidated (Fig. 1). The SAR analysis of oleanolic acid derivatives modified at C-3 and C-28 indicated that hydrogen-bond acceptor substitution at the C-3 position of oleanolic acid may be advantageous for the improvement of cytotoxicity against PC-3, A549 and MCF-7 cell lines [12]. Gnoatto found that the derivative with an acetylation at C-3 of the oleanolic acid backbone had much better activity against the *L. amazonensis* strain [17]. 3-Oxo oleanolic acid (3-oxo-olea-12-en-28-oic acid), a derivative of oleanolic acid modified at C-3, was found to significantly inhibit the growth of cancer cells derived from different tissues, particularly on melanoma in vivo [18]. Some other acyl compounds, generated from the modification of the hydroxyl groups of the A-ring in the triterpene skeleton of oleanolic acid and maslinic acid (MA, Fig. 1) with 10 different acyl groups, displayed cytotoxic effects against b16f10 murine melanoma cells and showed apoptotic effects with high levels of early and total apoptosis (up to 90%). These acyl compounds also exhibited better inhibition effects to anti-HIV-1-protease, with IC_50_ values between 0.31 and 15.6 μM, which are 4–186 times lower than their non-acylated precursors [19]. Compound **2** (Fig. 1), un benzyl (2α,3β) 2,3-diacetoxy-olean-12-en-28-amide, exhibited much better cytotoxicity against human tumor cell lines compared with its deacylation product, while it showed a rather low cytotoxicity for human fibroblasts (WW030272) [20].

On the other hand, pyrimidine has been widely used as an anti-tumor pharmacophore in medicinal chemical research [21]. For instance, some new pyridines and pyrazolo [1,5-α] pyrimidines exhibited potent anti-tumor cytotoxic activity in vitro against different human cell lines [22]. The evaluation of several ring-A fused hybrids of oleanolic acid against seven human cancer cell lines showed that the fused pyrimidine moiety seemed important to enhance the antiproliferative activity of oleanolic acid [23]. Thus, the pyrimidinyl group has been constructed in many new compounds in various anti-tumor studies [24].

## Results and discussion

### Synthesis

**Scheme 1:**
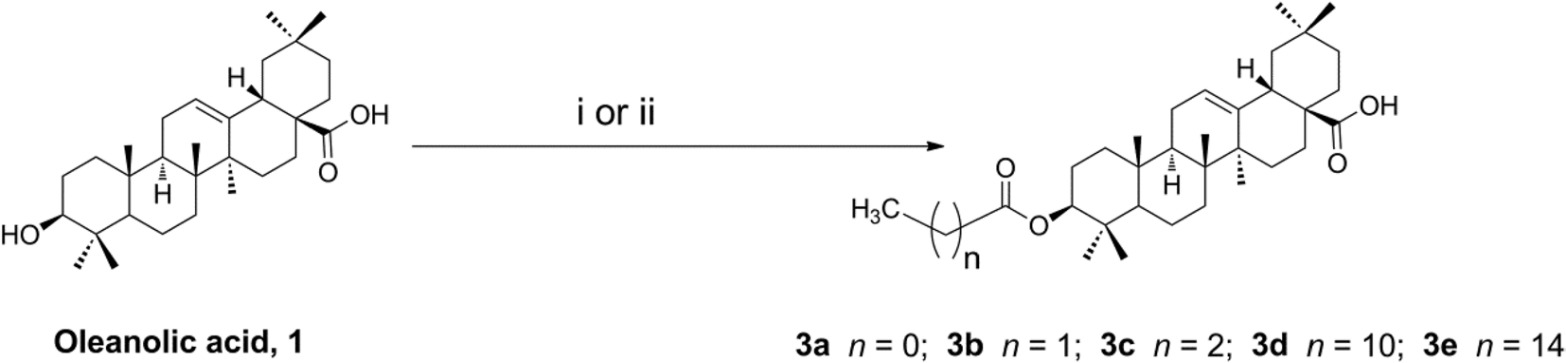
Synthesis of acyl oleanolic acid derivatives. Reagents and conditions: (i) anhydride, DMAP, anhydrous CH_2_Cl_2_/pyridine, rt (64–89%); (ii) acyl chloride, Et_3_N, anhydrous THF, rt (75–88%)

**Scheme 2:**
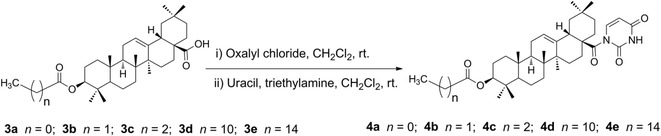
Synthesis of oleanolic acid-uracil conjugates, where n is the number of methylene groups in the acyl group

Inspired by the cited evidence, in this study, we conjugated five different acyl OA derivatives (**3a**–**3e**) [15, 19, 20, 25, 26] with uracil. The synthetic routes are outlined in Schemes 1 and 2. The treatment of **1** (1 equiv) with anhydride (1.5 equiv) and DMAP (0.1 equiv) in anhydrous CH_2_Cl_2_/pyridine (1/7 = v/v) at room temperature afforded 3-*O*-acyl derivatives **3a**–**3c** [15, 19, 20, 25] (64–89%). The treatment of **1** (1 equiv) with acyl chloride (3 equiv) and Et_3_N (3.5 equiv) in anhydrous THF at room temperature gave acyl derivatives **3d**–**3e** [19, 26] (75–88%). The acyl oleanolic acid compounds (**3a**–**3e**, 1 equiv) were then first treated with oxalyl chloride (18 equiv) to give the corresponding acyl chloride, which was then treated with uracil (3 equiv) in the presence of Et_3_N to generate the corresponding acyl oleanolic acid-uracil conjugates (**4a**–**4e**, Scheme 2) in 11–60% yields. The structures of compounds **4a**–**4e** were confirmed by NMR and mass spectra.

### Cytotoxicity

As anti-tumor effects are the most classical activities of oleanolic acid and its derivatives [1, 27–29], these conjugates have been evaluated by MTT assay [30, 31] against 5 adherent tumor cell lines (Hep-G2, A549, BGC-823, MCF-7 and PC-3) with **1** as the positive control. 5-Fluorouracil (5-FU), a medication used in the clinical treatment of cancer, is also a pyrimidine analog and was used as a positive control in this study. The results are presented in Table 1.

**Table 1:** Evaluation of **4a**–**4e** against different tumor cell lines

Compounds	IC_50_ (μM)^a^				
	Hep-G2	A549	BGC-823	MCF-7	PC-3
**4a**	7.83 ± 0.69	4.01 ± 0.37	<0.1	<0.1	NI^b^
**4b**	2.81 ± 0.22	15.5 ± 1.34	<0.1	6.53 ± 0.35	NI
**4c**	5.24 ± 0.05	3.01 ± 0.28	0.22 ± 0.02	6.99 ± 0.57	0.28 ± 0.05
**4d**	8.49 ± 0.68	0.27 ± 0.03	0.11 ± 0.01	3.51 ± 0.22	10.61 ± 1.13
**4e**	4.19 ± 0.35	8.76 ± 0.07	< 0.1	5.26 ± 0.41	2.51 ± 0.05
**1**	15.90 ± 1.13	16.29 ± 1.36	23.74 ± 1.53	12.60 ± 1.09	72.74 ± 6.88
**5-FU**	55.74 ± 5.09	22.62 ± 2.19	8.82 ± 0.78	21.47 ± 1.99	12.23 ± 1.18

^a^IC_50_ values are presented as the mean ± SD (standard deviation) from three separated experiments

^b^No inhibition detected

The results showed that these compounds exhibited excellent antiproliferative activities against the tested cells, with the IC_50_ values mainly under 10.0 μM, except for compounds **4a** and **4b** which showed no inhibition against the PC-3 cell line. In the Hep-G2, A549, BGC-823 and MCF-7 cell line assays, all the synthesized compounds displayed much better inhibition than that of **1** and 5-FU. With a propionyloxy group at C-3, compound **4b** possessed the best inhibition activity against the Hep-G2 cell line, almost 5.5-fold and 20-fold stronger than **1** and 5-FU, respectively. With a dodecanoyloxy group at C-3, compound **4d** showed the best inhibition activity against the A549 cell line, almost 60-fold and 84-fold stronger than **1** and 5-FU, respectively. Meanwhile, compound **4a**, with an acetoxy group at C-3, exhibited the best inhibition activity against the MCF-7 cell line, more than 126-fold and 215-fold more effective than **1** and 5-FU respectively. Compounds **4a** (acetoxy), **4b** (propionyloxy) and **4e** (palmitoxy) exhibited excellent antiproliferative activities against the BGC-823 cell line (IC_50_ < 0.1 μM). Although compounds **4a** (acetoxy) and **4b** (propionyloxy) possessed good antiproliferative activities against the Hep-G2, A549, BGC-823 and MCF-7 cell lines, they showed no inhibition against the PC-3 cell line. In the PC-3 assay, the butyryloxy compound **4c** exhibited the best antiproliferative activity, being 260-fold and 44-fold stronger than **1** and 5-FU, respectively. The results above reveal that in general, the acyl groups at the C-3 position of these uracil conjugates have primarily made a great contribution to the antiproliferative activities against the tested cell lines.

For further analysis, conjugate **4c** was selected to determine its cytotoxicity selectivity and mechanism of growth inhibition on an adherent Hep-G2 cell line. The controls of the figures were reused from our previous work [32].

### Cytotoxicity selectivity

**Fig. 2:**
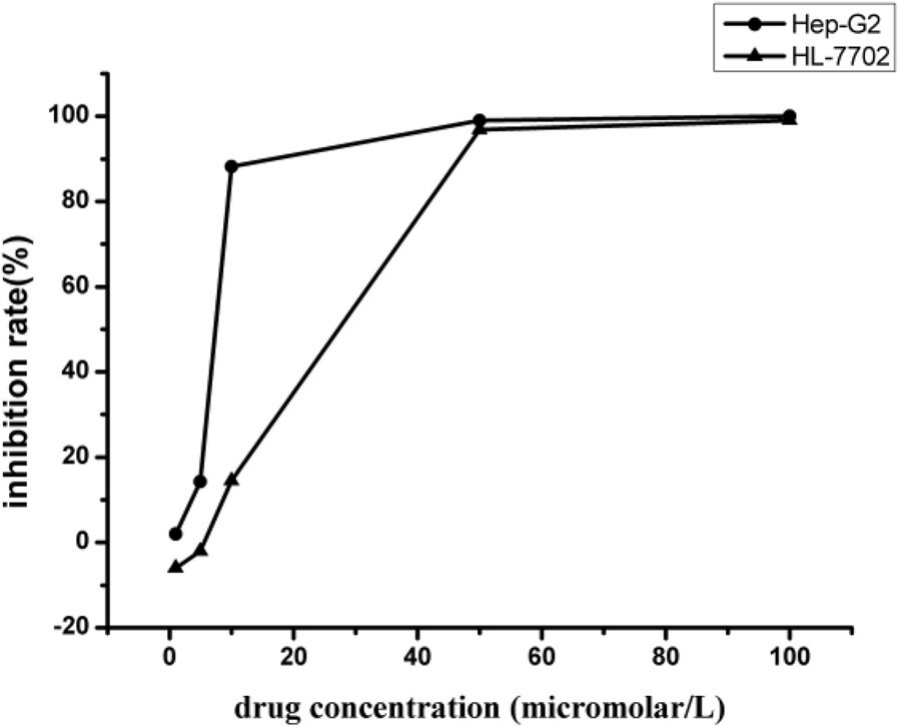
Cytotoxicity of **4c** against Hep-G2 tumor cells and HL-7702 human liver cells. Hep-G2 and HL-7702 cells were cultured in medium in the presence of the indicated concentrations of **4c** for 72 h

As shown in Fig. 2, though the inhibition rate of **4c** against human liver cell line HL-7702 (L-O2) at the concentration of 50 μM was equivalent to that of human hepatoma cell line Hep-G2, its inhibition rate against the HL-7702 cell line was only approximately 15% at the concentration of 10 μM, while the inhibition rate against the Hep-G2 cell line was up to 90% at the same concentration. Thus, it was exhibited that compound **4c** showed strong cytotoxicity selectivity to human hepatocellular carcinoma cells in vitro at the therapeutically effective concentration.

### Fluorescence staining

**Fig. 3:**
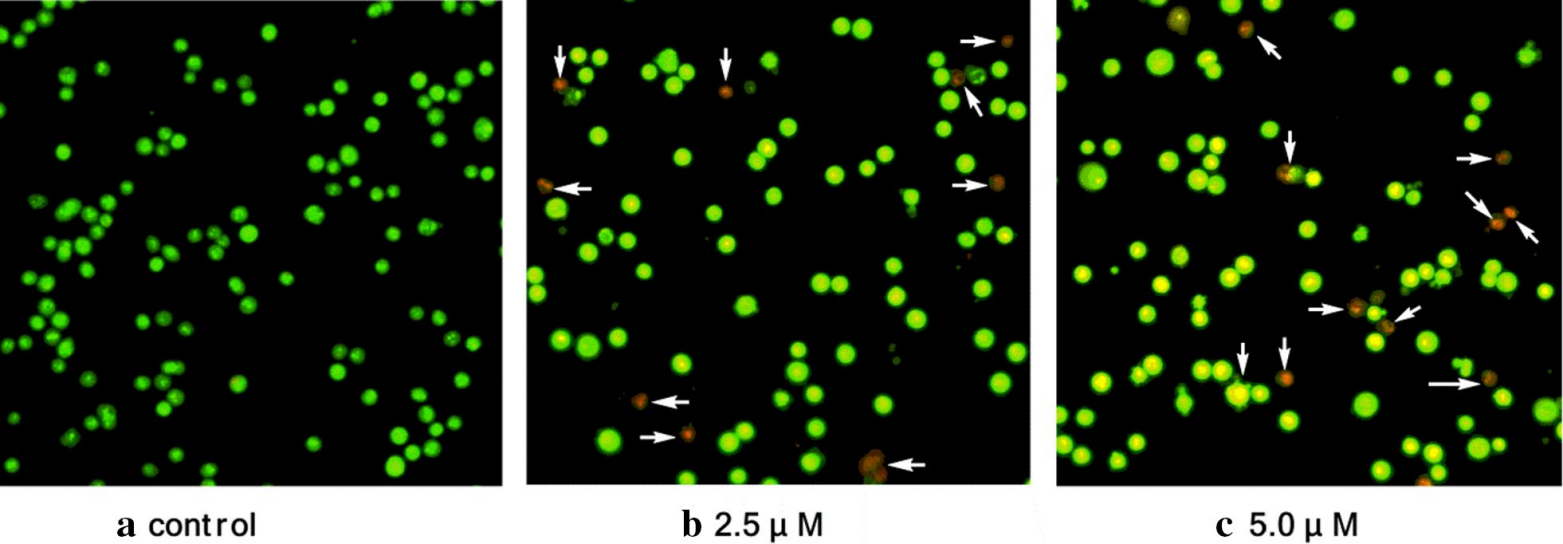
Cell morphological observation for cell apoptosis induction on the Hep-G2 cells treated by **4c**. **a** Control cells; **b, c** cells treated by **4c** for 24 h; cells were stained by AO/EB, and selected visual fields illustrating the corresponding live cells, early apoptotic cells (*white arrow*) are shown (magnification ×200)

**Fig. 4:**
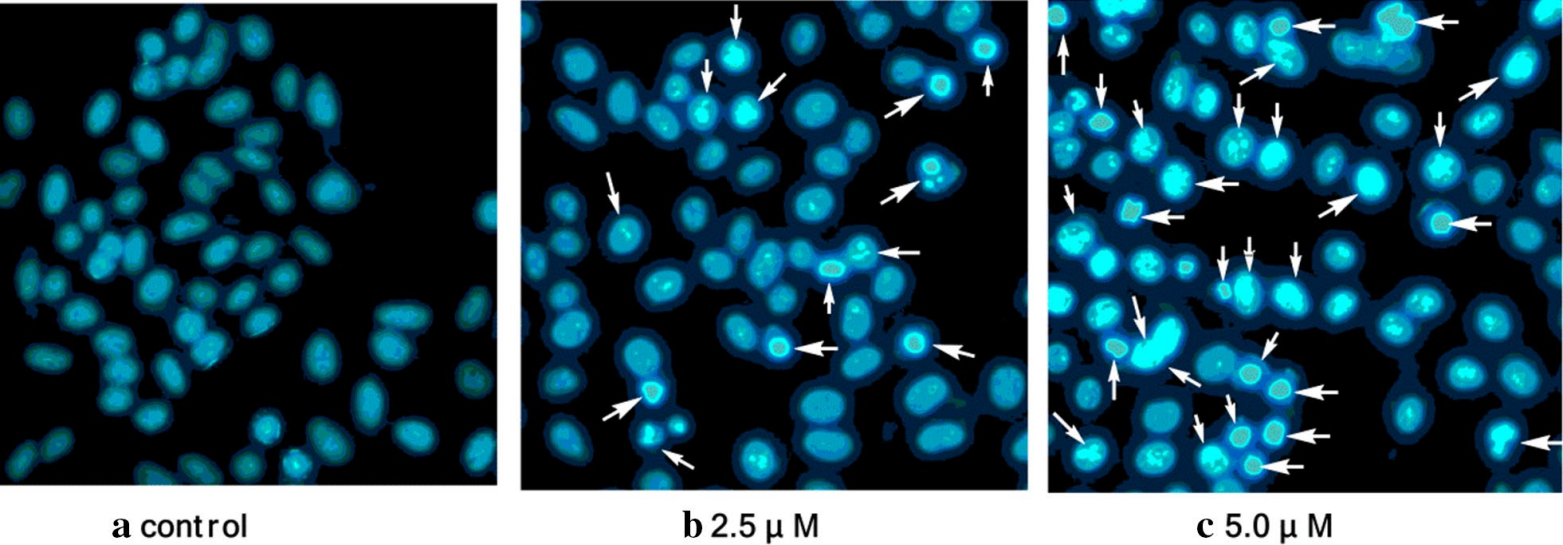
Cell morphological observation for cell apoptosis induction on the Hep-G2 cells treated by conjugate **4c**. **a** Control cells; **b, c** cells treated by **4c** for 24 h; cells were stained by Hoechst 33258, and selected visual fields illustrating the condensed chromatin (*white arrow*) as occurrence of cell apoptosis are shown (magnification ×200)

After sequentially staining with acridine orange (AO)/ethidium bromide (EB) (Fig. 3) and Hoechst 33258 (Fig. 4), the living cells were treated with compound **4c** (2.5 and 5.0 μM, 24 h). As depicted in Fig. 3, the living cells excluded EB and staining by AO caused a green color (Fig. 3a), whereas the Hep-G2 cells treated with **4c** had obviously changed (Fig. 3b, c). Under fluorescence microscopy, early apoptosis cells were observed to emit orange or dark orange fluorescence, with nuclear morphological changes, which suggested that **4c** could induce apoptosis in Hep-G2 cells. This is consistent with the results of Hoechst 33258 staining shown in Fig. 4. The nuclei of the Hep-G2 cells retained the regular round contours in the control group (Fig. 4a), and cells with smaller nuclei and condensed chromatin were rarely observed. It was found that the contours of some of the Hep-G2 cells became irregular even when they were exposed to **4c** at lower concentration of 2.5 μM, accompanied with the nuclei being condensed (as the bright blue fluorescence indicates) and the apoptotic bodies appeared (Fig. 4b). When treated with **4c** at a higher concentration of 5.0 μM, the nuclei of many more cells were highly condensed and the apoptotic bodies were pervasive in the visual field (Fig. 4c). These clear changes to the cell morphology suggested the significant cell apoptosis induction of **4c** on Hep-G2 cells.

### Cell cycle analysis

**Fig. 5:**
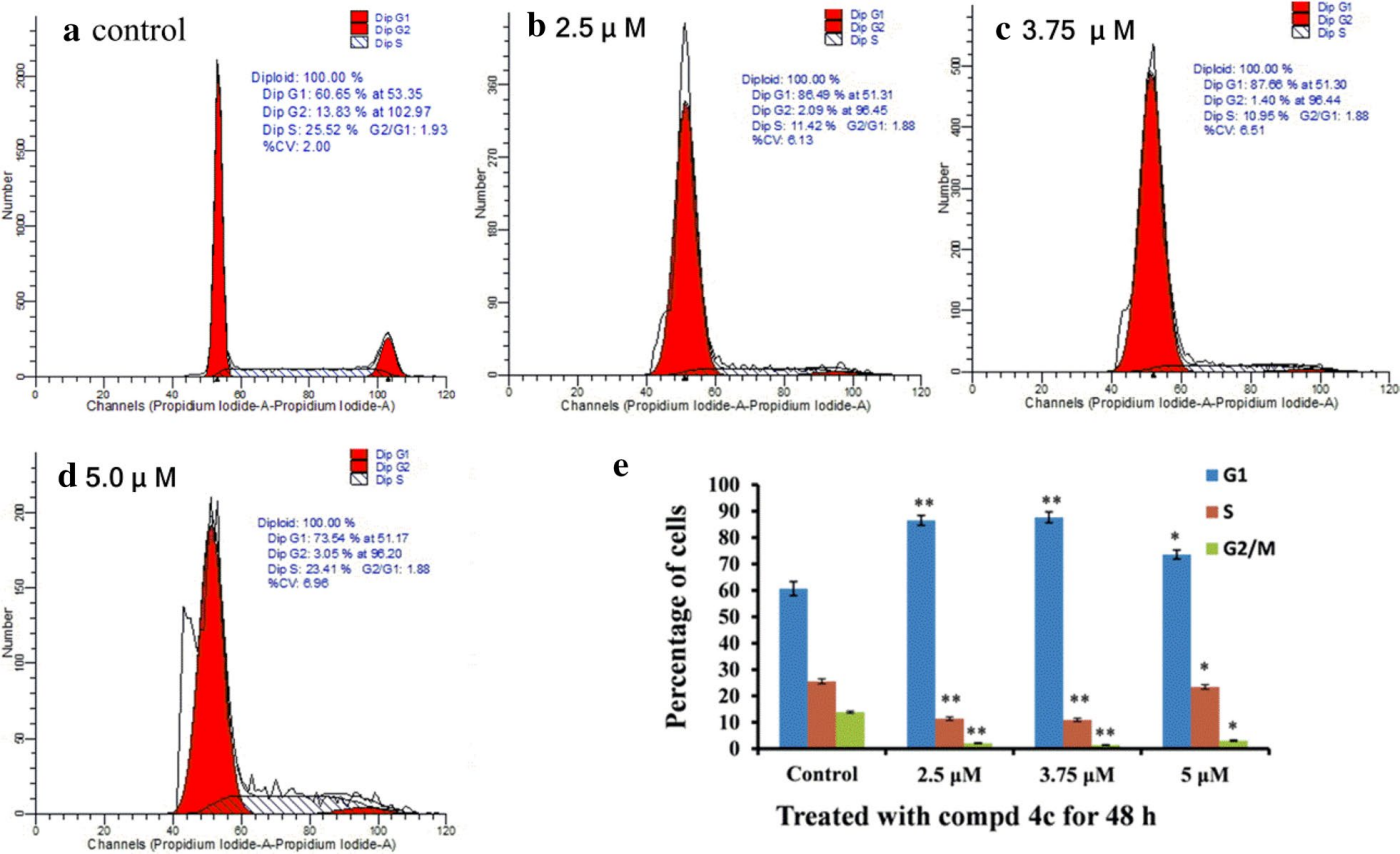
Cell cycle progress detection by flow cytometry assay after PI staining of Hep-G2 cells following treatment with compound **4c**: **a** control cells; **b, c, d** cells treated by **4c** for 48 h; **e** corresponding histograms. *P < 0.05 and **P < 0.01 compared with the control

To confirm whether the decrease of cell viability was caused by cell cycle arrest, the Hep-G2 cells were treated with compound **4c** for 48 h at different concentrations and then the cell cycle distribution was determined by a flow cytometry assay after propidium iodide (PI) staining (Fig. 5). The results indicate that conjugate **4c** enhanced the cell cycle arrest at the G1 phase at different concentrations, resulting in a concomitant population increase in the G1 phase (60.65–86.49, 87.66 and 73.54%), and declines in the cell population in the G2/M (13.83–2.09, 1.40 and 3.05%) and S-phases (25.52–11.42, 10.95 and 23.41%).

### AnnexinV/propidium iodide assay

**Fig. 6:**
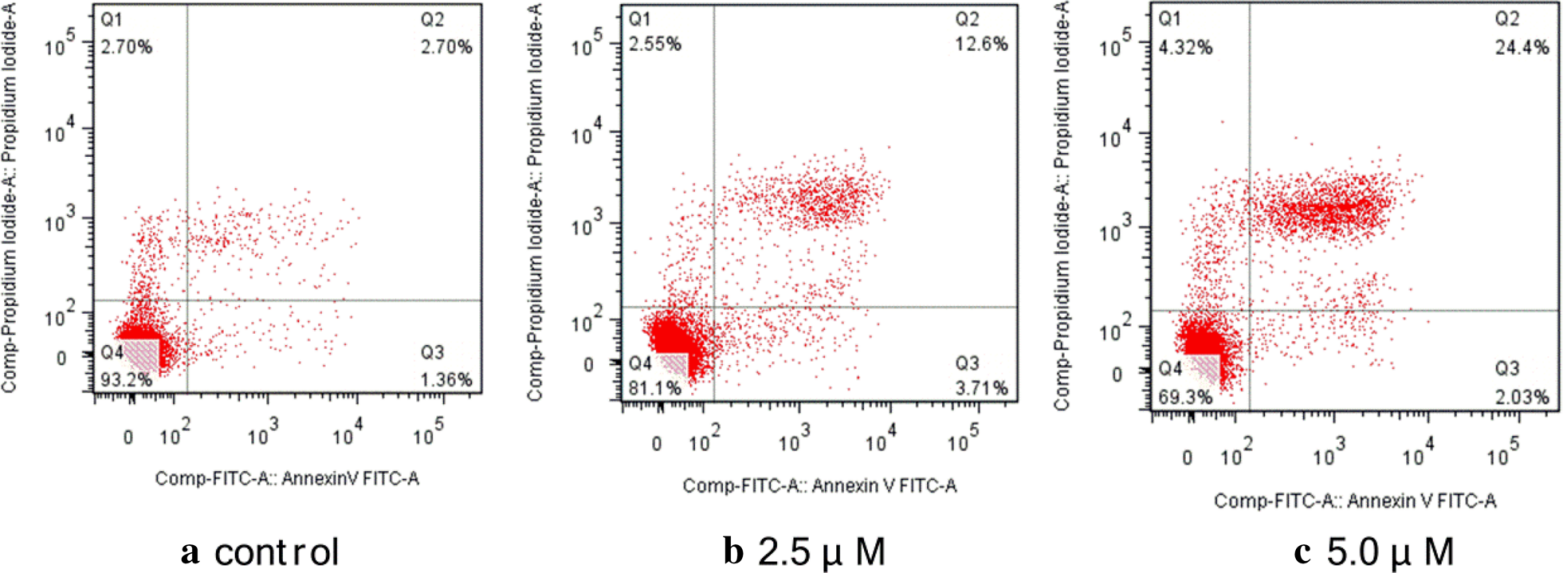
Apoptosis ratio detection by Annexin V/PI assay on the Hep-G2 cells treated by conjugate **4c**: **a** control cells; **b, c** cells treated by **4c** for 24 h

To determine whether the observed cell death induced by conjugate **4c** was due to apoptosis or necrosis, the interactions of Hep-G2 cells with **4c** were further investigated using an Annexin V-FITC/PI assay (Fig. 6). The apoptosis ratios (including the early and late apoptosis ratios) of **4c** measured at different concentration points were found to be 16.31% (2.5 μM) and 26.43% (5.0 μM), respectively, while that of the control was 4.06%. This revealed that **4c** could mainly induce later period apoptosis in Hep-G2 cells.

### Mitochondrial membrane potential detection

**Fig. 7:**
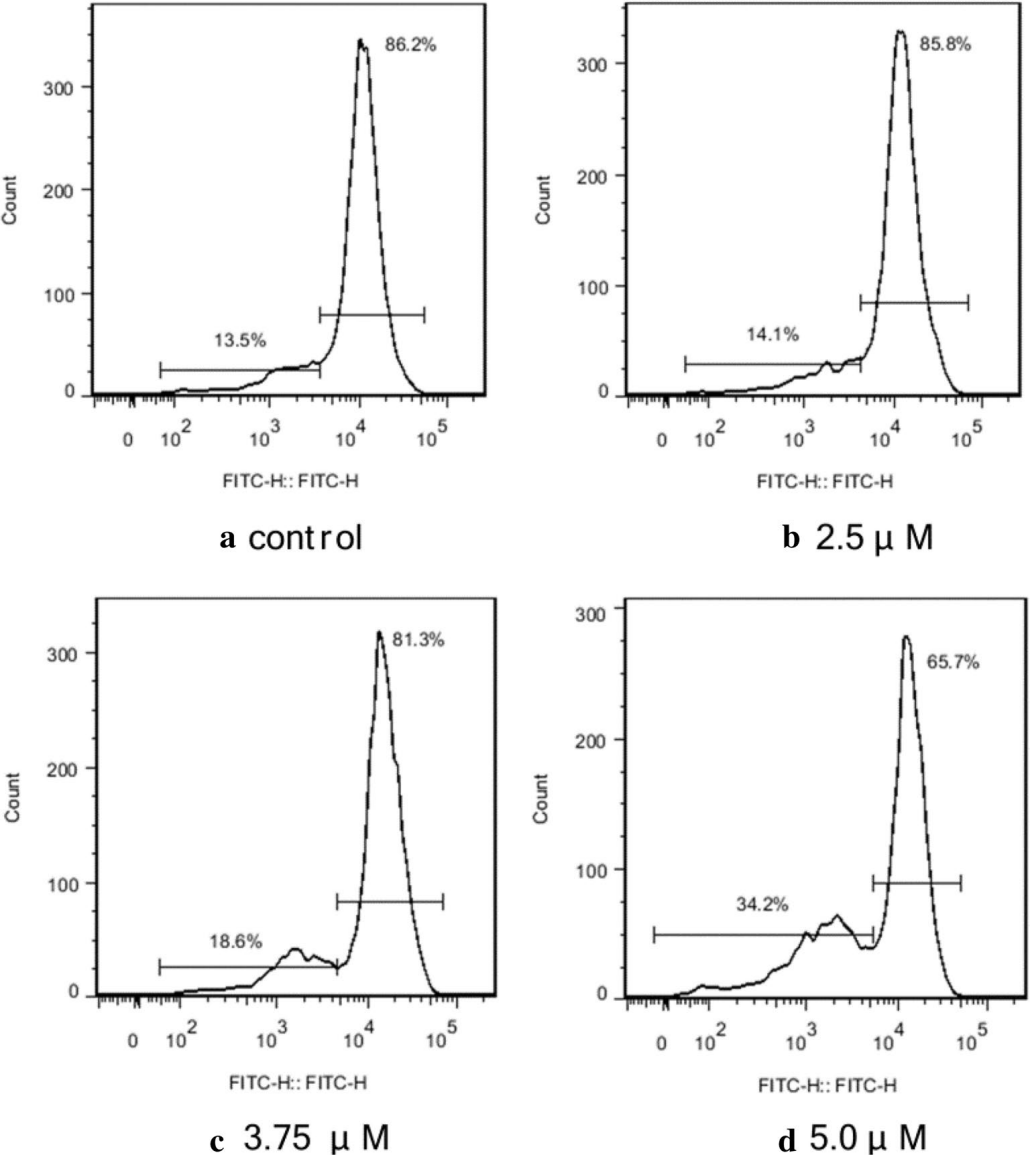
Collapse of mitochondrial membrane potential in the Hep-G2 cells treated by conjugate **4c**. **a** Control cells; **b, c, d** cells treated by **4c** for 24 h; cells were stained with Rhodamine 123 for 30 min. The results are expressed as relative fluorescent intensity

As the mitochondrial membrane potential (Δψ) has been considered a new antitumor target [33, 34], the changes in Δψ in Hep-G2 cells (treated with conjugate **4c**) stained with Rhodamine 123 indicated by flow-cytometric analysis were tested (Fig. 7). The results indicated that **4c** induced a marked concentration-dependent decrease of Rhodamine 123 fluorescence (decreasing from 86.2% to 85.8, 81.3 and 65.7% with the increase concentration of **4c**). This indicated that compound **4c** can induce mitochondrial membrane potential disruption in Hep-G2 cells.

### Caspase-3/9 activity assay

**Fig. 8:**
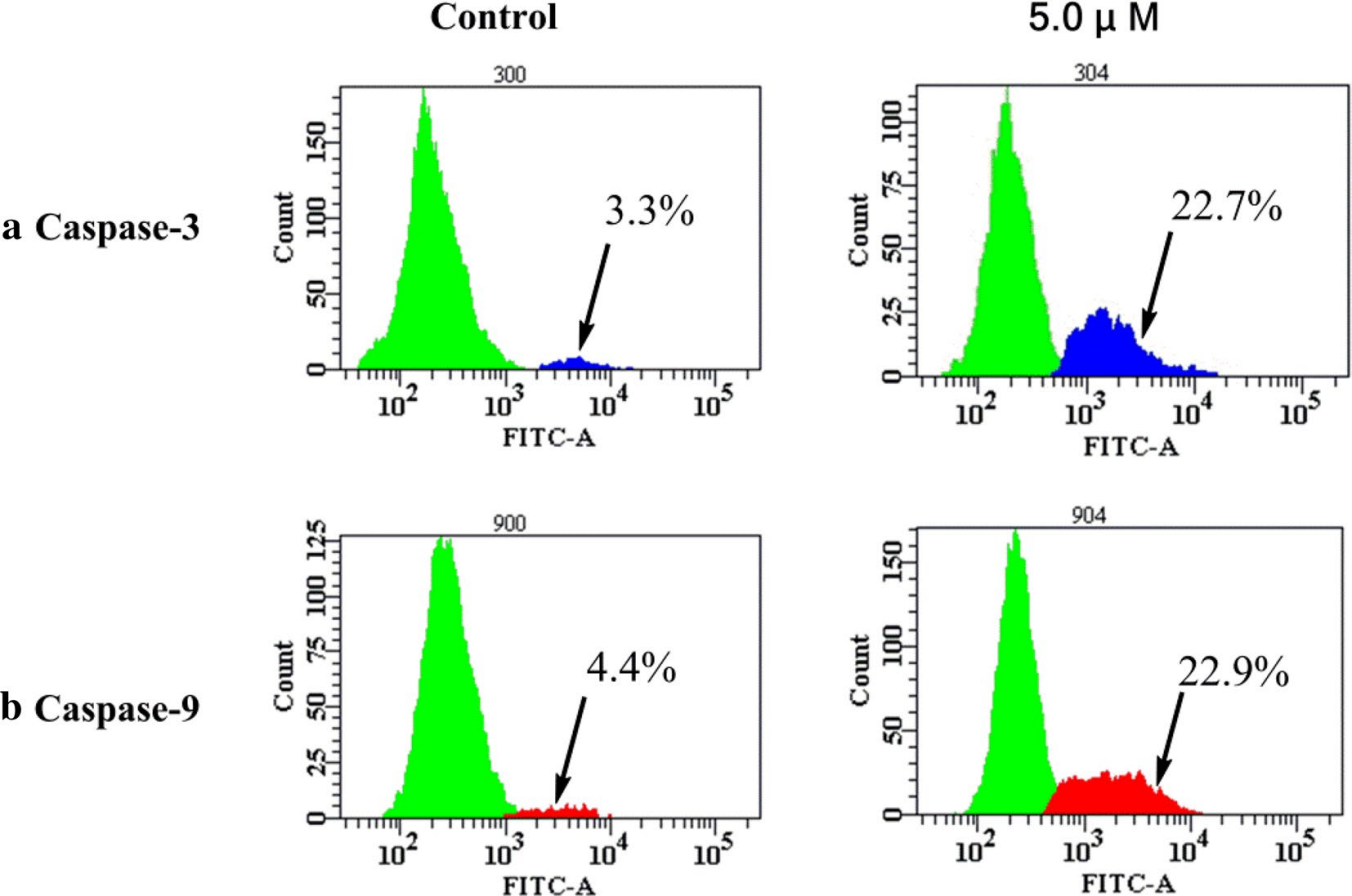
Activation of caspase-3/9 by conjugate **4c** in Hep-G2 cells after treatment with 5.0 μM for 24 h: **a** activation of caspase-3; **b** activation of caspase-9

FITC-DEVD-FMK (for caspase-3) and FITC-LEHD-FMK (for caspase-9) probe assays were carried out to determine the death signaling in the caspase family after treatment with **4c** (5.0 μM, 24 h) in Hep-G2 cells. The proportion of activated-caspase-3 cells after **4c** treatment was enhanced to 22.7% (Fig. 8a), while that of activated-caspase-9 cells was enhanced to 22.9% (Fig. 8b). These results indicate that **4c** could induce cell apoptosis by triggering caspase-3/9 activity in Hep-G2 cells.

## Conclusions

Five acyl oleanolic acid-uracil conjugates were synthesized and their anti-tumor activities were evaluated. These conjugates exhibited excellent antiproliferative activities against the tested cells (Hep-G2, A549, BGC-823, MCF-7 and PC-3) except compounds **4a** and **4b**, which showed no inhibition against the PC-3 cell line. Most of the IC_50_ values were under 10.0 μM, with some of them even under 0.1 μM.

Conjugate **4c** was selected for further analysis including for its cytotoxicity selectivity and its mechanism of growth inhibition on the Hep-G2 cell line. The inhibition rate of **4c** against the HL-7702 cell line was only approximately 15% (90% against Hep-G2) at the concentration of 10 μM, indicating that it had strong cytotoxicity selectivity to human hepatocellular carcinoma cells in vitro. The treatment of Hep-G2 cells with this compound, could induce changes in the permeability of the mitochondrial membrane, and thus cause a decline in the mitochondrial membrane potential. With the disruption of the mitochondrial membrane potential, changes in cellular morphology appeared as a result of significant apoptosis induction. Then, cell proliferation in the G1 phase was arrested and apoptotic signaling activated caspase-9. Caspase-9, as a protease, can activate the apoptotic effector caspase-3, eventually causing nuclear apoptosis. Further studies of the specific mechanisms of these compounds in human malignant tumors are currently underway.

## Methods

### General

All commercially available solvents and reagents used were of analytical grade and were used without further purification. All commercial reagents were purchased from Aladdin (Shanghai) Industrial Corporation. Melting points were measured on a RY-1 melting point apparatus. ^1^H and ^13^C-NMR spectra were recorded on Bruker AV-500 (500/125 MHz for ^1^H/^13^C) spectrometers. Chemical shifts are reported as values relative to an internal tetramethylsilane standard. The low-resolution mass spectra were obtained on a Bruker Esquire HCT spectrometer, and HRMS were recorded on a Thermo Scientific Accela—Exactive High Resolution Accurate Mass spectrometer.

### Chemistry

#### General procedures for synthesis of oleanolic acid-uracil conjugates 4a–4e

To a solution of different acyl oleanolic acid compound (**3a**–**3e**, 0.2 mmol) in anhydrous CH_2_Cl_2_ (3 mL) at 0 °C, oxalyl chloride (0.34 mL, 3.6 mmol) was added. After stirring at room temperature for 12 h, the mixture was evaporated, and co-evaporated with CH_2_Cl_2_ (3 × 1 mL). The residue was dissolved in dry THF (3 mL), and then Et_3_N (1 mL, 0.7 mmol) and uracil (0.067 g, 0.6 mmol) were added at 0 °C. After stirring at r.t. for 24 h, the solvent was evaporated. The residue was then taken up in H_2_O (35 mL) and extracted with CH_2_Cl_2_ (3 × 20 mL). The combined organic layers were washed with H_2_O and brine, dried with MgSO_4_, filtered and concentrated to give a crude product. The crude product was purified by flash column chromatography to afford the corresponding product (**4a**–**4e**) respectively (Additional file 1).

#### Compound **4a**

Compound **4a** was prepared from **3a** [15, 19, 20] (1.000 g, 2.00 mmol) and uracil (0.673 g, 6.00 mmol) according to the general procedure. The residue was purified by flash column chromatography (Petroleum ether:EtOAc = 4: 1). Yield: 0.349 g, 29%, white solid, mp 209–211 °C. ^1^H NMR (500 MHz, CDCl_3_) δ 0.69, 0.85, 0.86, 0.99 and 1.17 (5 s, each 3H, 5 × CH_3_), 0.92 (s, 6H, 2 × CH_3_), 0.63–2.15 (m, 22H), 2.05 (s, 3H, CH_3_COO), 2.99 (dd, 1H, *J* = 3.2, 13.2 Hz, H-18), 4.48 (dd, 1H, *J* = 6.6, 9.3 Hz, H-3), 5.28 (t, 1H, *J* = 3.2 Hz, H-12), 5.74 (d, 1H, *J* = 8.3 Hz, H-5^*Ura*^), 7.51 (d, 1H, *J* = 8.3 Hz, H-6^*Ura*^), 8.20 (brs, 1H, NH). ^13^C NMR (125 MHz, CDCl_3_) δ 15.6, 16.8, 18.2, 18.3, 21.4, 23.0, 23.7, 24.0, 26.1, 27.7, 28.2, 30.0, 30.7, 32.7, 33.2, 33.9, 37.0, 37.9, 38.4, 39.8, 42.0, 43.2, 46.5, 47.5, 53.3, 55.3, 81.0, 103.0, 123.6, 141.7, 143.3, 148.8, 162.7, 171.2, 182.0. HRMS (ESI)* m*/*z*: [M−H]^+^ calcd for C_36_H_51_N_2_O_5_, 591.3798; found 591.3808.

#### Compound **4b**

Compound **4b** was prepared from **3b** [19, 25] (0.300 g, 0.59 mmol) and uracil (0.196 g, 1.75 mmol) according to the general procedure. The residue was purified by flash column chromatography (Petroleum ether:EtOAc = 5: 1). Yield: 0.213 g, 60%, white solid, mp 119–121 °C. ^1^H NMR (500 MHz, CDCl_3_) *δ* 0.69, 0.84, 0.85, 0.98 and 1.17 (5 s, each 3H, 5 × CH_3_), 0.92 (s, 6H, 2 × CH_3_), 1.14 (t, *J* = 7.6 Hz, CH_3_), 0.63–2.13 (m, 22H), 2.32 (q, 2H, *J* = 7.3 Hz, CH_2_COO), 2.99 (dd, 1H, *J* = 2.5, 12.8 Hz, H-18), 4.48 (dd, 1H, *J* = 6.5, 9.3 Hz, H-3), 5.28 (s, 1H, H-12), 5.74 (d, 1H, *J* = 8.3 Hz, H-5^*Ura*^), 7.50 (d, 1H, *J* = 8.3 Hz, H-6^*Ura*^), 8.97 (brs, 1H, NH). ^13^C NMR (125 MHz, CDCl_3_) *δ* 9.4, 15.6, 16.9, 18.1, 18.2, 22.9, 23.6, 24.0, 26.1, 27.7, 28.1, 28.2, 30.0, 30.6, 32.7, 33.1, 33.9, 37.0, 37.9, 38.4, 39.8, 42.0, 43.2, 46.5, 47.5, 53.2, 55.3, 80.6, 103.0, 123.6, 141.7, 143.3, 149.0, 163.4, 174.4, 182.0. HRMS (FTMS + pESI)* m*/*z*: [M−H]^+^ calcd for C_37_H_53_N_2_O_5_, 605.3955; found 605.3979.

#### Compound **4c**

Compound **4c** was prepared from **3c** [19, 25] (0.200 g, 0.38 mmol) and uracil (0.127 g, 1.14 mmol) according to the general procedure. The residue was purified by flash column chromatography (Petroleum ether:EtOAc = 3: 1). Yield: 0.131 g, 56%, white solid, mp 285–287 °C. ^1^H NMR (500 MHz, CDCl_3_) *δ* 0.69, 0.85, 0.86, 0.95, 0.99 and 1.18 (6 s, each 3H, 6 × CH_3_), 0.92 (s, 6H, 2 × CH_3_), 0.63–2.15 (m, 24H), 2.28 (t, 2H, *J* = 7.1 Hz, CH_2_COO), 2.99 (dd, 1H, *J* = 3.3, 13.2 Hz, H-18), 4.49 (dd, *J* = 5.7, 10.1 Hz, 1H, H-3), 5.28 (s, 1H, H-12), 5.74 (d, 1H, *J* = 8.3 Hz, H-5^*Ura*^), 7.50 (d, 1H, *J* = 8.3 Hz, H-6^*Ura*^), 8.30 (brs, 1H, NH). ^13^C NMR (100 MHz, CDCl_3_) 13.7, 15.4, 16.8, 17.2, 18.2, 18.6, 23.4, 23.6, 25.7, 25.8, 27.8, 28.0, 30.7, 32.0, 32.7, 33.0, 33.8, 36.8, 36.9, 37.7, 38.1, 40.0, 41.3, 41.9, 45.8, 47.4, 47.5, 55.3, 80.5, 104.5, 112.3, 123.0, 139.6, 142.9, 149.0, 161.8, 173.5, 175.2. APCI-MS* m*/*z*: 619.4 [M−H]^+^. HRMS (ESI)* m*/*z*: [M−H]^+^ calcd for C_38_H_55_N_2_O_5_, 619.4111; found 619.4113.

#### Compound **4d**

Compound **4d** was prepared from **3d** [19] (0.280 g, 0.44 mmol) and uracil (0.148 g, 1.32 mmol) according to the general procedure. The residue was purified by flash column chromatography (Petroleum ether:EtOAc = 5: 1). Yield: 0.072 g, 22%, white solid. ^1^H NMR (500 MHz, CDCl_3_) *δ* 0.70, 1.00 and 1.19 (3 s, each 3H, 3 × CH_3_), 0.87 and 0.93 (2 s, each 6H, 4× CH_3_), 0.63–2.10 (m, 43H), 2.30 (s, 2H, CH_2_COO), 3.01 (d, 1H, *J* = 11.8 Hz, H-18), 4.50 (s, 1H, H-3), 5.30 (s, 1H, H-12), 5.74 (d, 1H, *J* = 7.5 Hz, H-5^*Ura*^), 7.52 (d, 1H, *J* = 7.7 Hz, H-6^*Ura*^), 8.72 (brs, 1H, NH). ^13^C NMR (125 MHz, CDCl_3_) *δ* 14.3, 15.6, 16.9, 18.1, 18.2, 22.8, 23.0, 23.6, 24.0, 25.3, 26.1, 27.7, 28.2, 29.3, 29.4, 29.5, 29.6, 29.7, 30.0, 30.7, 32.1, 32.6, 33.2, 33.9, 35.0, 37.0, 37.9, 38.4, 39.8, 42.0, 43.2, 46.5, 47.5, 53.2, 55.3, 80.5. 103.0, 123.6, 141.7, 143.3, 148.9, 163.1, 173.9, 182.0. HRMS (ESI)* m*/*z*: [M−H]^+^ calcd for C_46_H_71_N_2_O_5_, 731.5363; found 731.5387.

#### Compound **4e**

Compound **4e** was prepared from **3e** [26] (0.347 g, 0.50 mmol) and uracil (0.168 g, 1.50 mmol) according to the general procedure. The residue was purified by flash column chromatography (Petroleum ether:EtOAc = 5: 1). Yield: 0.044 g, 11%, white solid, mp 89–91 °C. ^1^H NMR (500 MHz, CDCl_3_) δ 0.69, 0.85, 0.86, 0.99 and 1.18 (5 s, each 3H, 5× CH_3_), 0.92 (s, 6H, 2× CH_3_), 0.63–2.10 (m, 51H), 2.29 (t, 2H, *J* = 7.5 Hz, CH_2_COO), 2.99 (dd, 1H, *J* = 2.7, 12.9 Hz, H-18), 4.48 (dd, 1H, *J* = 5.9, 9.8 Hz, H-3), 5.28 (s, 1H, H-12), 5.74 (d, 1H, *J* = 8.3 Hz, H-5^*Ura*^), 7.50 (d, 1H, *J* = 8.3 Hz, H-6^*Ura*^), 8.17 (brs, 1H, NH). APCI-MS* m*/*z*: 787.7 [M−H]^+^. HRMS (ESI)* m*/*z*: [M−H]^+^ calcd for C_50_H_79_N_2_O_5_, 787.5989; found 787.6003.

### Cell lines and culture

The human hepatocellular cell line Hep-G2, human lung cancer cell line A549, human gastric tumor cell line BGC-823, human breast tumor cell line MCF-7, human prostate cancer cell line PC-3 and human hepatocyte cell line HL-7702, these adherent cells were purchased from the Cell Bank of Type Culture Collection of the Chinese Academy of Sciences (Shanghai) and cultured in DMEM medium supplemented with 10% FCS (Fetal Calf Serum). The cells were incubated in an atmosphere of 5% CO_2_ and 95% air at 37 °C.

### MTT assay

The MTT assay was carried out according to a description in a published study [30, 31]. Cells were seeded in 96-well plates and incubated in a CO_2_ incubator at 37 °C. The tested compounds were dissolved in fresh culture medium with 2% DMSO to afford various concentrations (100, 50, 10, 5, 1 or 0.1 μmol/L). When the cells adhered, compounds at different concentrations were added to every well. The control wells contained medium supplemented with 2% DMSO. After incubation for another 72 h, 20 μL MTT (5%) was added to each well, and the cells were incubated for an additional 4 h at 37 °C. At last, the medium was removed carefully and dimethyl sulfoxide (100 μL) was added to each well. Then the plate was kept on a shaker for 10 min to mix these solutions properly. The absorbance of each well was scanned with an electrophotometer at 570 nm. Each concentration treatment was performed in triplicate wells. The IC_50_ values were estimated by fitting the inhibition data to a dose-dependent curve using a logistic derivative equation.

### AO/EB staining

This assay was carried out according to a description in a published study [35]. Cells were seeded at a concentration of 5 × 10^4^ cell/mL in a volume of 2 mL on a sterile cover slip in six-well tissue culture plates. Following incubation, the RPMI 1640 medium was removed and replaced with fresh medium plus 10% FCS and supplemented with compound **4c** at the indicated concentration. After the treatment period (24 h), the cover slip with monolayer cells was inverted on a glass slide with 20 μL of AO/EB stain (100 mg/mL). The fluorescence was read on a Nikon ECLIPSETE2000-S fluorescence microscope (Japan).

### Hoechst 33258 staining

This assay was carried out according to a description in a published study [35]. Cells grown on a sterile cover slip in six-well tissue culture plates were treated with compound for a certain range of time. The culture medium containing compounds was removed, and the cells were fixed in 4% paraformaldehyde for 10 min. After being washed twice with PBS, the cells were stained with 0.5 mL of Hoechst 33258 (0.5 μg/mL, Beyotime) for 5 min and then again washed twice with PBS. The stained nuclei were observed under a Nikon ECLIPSETE2000-S fluorescence microscope using 350 nm excitation and 460 nm emission.

### Mitochondrial membrane potential measurement

This assay was carried out as described in a published study [34]. The depolarization of the mitochondrial membrane potential for cell apoptosis results in the loss of Rhodamine123 from the mitochondria and a decrease in the intracellular fluorescence intensity. Prepared Hep-G2 cells were harvested and washed twice in cold PBS and then resuspended in Rhodamine 123 (2 μM) for 30 min in the dark. The fluorescence was measured by flow cytometry with an excitation wavelength of 485 nm and emission wavelength of 530 nm.

### Flow cytometric analysis of cell cycle and apoptosis

This assay was carried out according to a description in a published study [35]. The induced apoptosis was assayed by the Annexin V-FITC Apoptosis Detection kit (Beyotime, China), according to the manufacturer’s instructions. Briefly, the prepared Hep-G2 cells (1 × 10^6^ cells/mL) were washed twice with ice-cold PBS and then resuspended gently in 500 μL of binding buffer. Thereafter, the cells were stained in 5 μL of Annexin V-FITC and shaken well. Finally, the cells were mixed with 5 μL of PI, incubated for 20 min in the dark and subsequently analyzed using an FACS AriaII (Becton–Dickinson).

### Determination of caspase-3 and caspase-9 activities by flow cytometric analysis

According to a description in a published study [35], the measurement of the caspase-3 and caspase-9 activities was performed by a CaspGLOW™ Fluorescein Active Caspase-3 and Caspase-9 Staining Kit. The prepared Hep-G2 cells were harvested at a density of 1 × 10^6^ cells/mL in RPMI 1640 medium supplemented with 10% FCS. A total of 300 μL each from the induced and control cultures were incubated with 1 μL of FITC-DEVD-FMK (caspase-3) or FITC-LEHD-FMK (caspase-9) for 1 h in a 37 °C incubator with 5% CO_2_. Flow cytometric analysis was performed using a FACS AriaII flow cytometer (Becton–Dickinson) equipped with a 488 nm argon laser.

### Statistical analysis

The experiments were repeated three times, and the results were presented as mean ± standard deviation (SD). Student’s *t* test was used to process the statistical significance and the differences between groups with *P* < 0.05 were considered significant.

## Additional file

**Additional file 1.** The copies of NMR spectra of compounds **4a**–**4e**

## Abbreviations

A549: human alveolar adenocarcinoma cell line
Annexin V-FITC: apoptosis detection kit
AO/EB: acridine orange/ethidium bromide
BGC-823: human grastic cancer cell line
CH_2_Cl_2_: dichloromethane
CDDO: 2-cyano-3,12-dioxooleana-1,9-dien-28-oic acid
CDDO-Im: 2-cyano-3,12-dioxooleana-1,9-dien-28-imidazolide
CDDO-Me: 2-cyano-3,12-dioxooleana-1,9-dien-28-oic acid methyl ester
DMAP: 4-dimethylaminopyridine
DMEM: dulbecco’s modified eagle’s medium
DMSO: dimethyl sulfoxide
Et_3_N: triethylamine
EtOAc: ethyl acetate
5-FU: 5-fluorouracil
FCS: fetal calf serum
FITC-DEVD-FMK: caspase-3 detection kit
FITC-LEHD-FMK: caspase-9 detection kit
G1 phase: gap1, pre-synthetic period
G2/M: gap2, post-synthetic period/mitosis
H_2_O: water
Hep-G2: human hepatoma cell line
HL-7702 (L-O2): hepatic immortal cell line
HRMS: high resolution mass spectrometry
IC_50_: half maximal inhibitory concentration
MA: maslinic acid
MCF-7: human breast adenocarcinoma cell line
MgSO_4_: magnesium sulfate
MTT: 3-(4, 5-dimethyl-2-thiazolyl)-2,5-diphenyl-2-H-tetrazolium bromide
NMR: nuclear magnetic resonance
OA: oleanolic acid
PBS: phosphate buffered saline
PC-3: human prostatic carcinoma cell line
PI: propidium iodide
RPMI: roswell park memorial institute
SAR: structure–activity relationships
SD: standard deviation
S-phase: synthetic period
TCM: traditional Chinese medicines
THF: tetrahydrofuran
Δψ: the mitochondrial membrane potential
μM: micromolar
