# Supplementary material for: Synthesis of acyl oleanolic acid-uracil conjugates and their anti-tumor activity
Source: Chem Cent J. 2016 Nov 21;10:69. doi: 10.1186/s13065-016-0217-5 (PMC5117584; doi:10.1186/s13065-016-0217-5)

Supporting Information

Synthesis of acyl oleanolic acid-uracil conjugates and their anti-tumor activity

Wei-bin Mo^1,3,†^, Chun-hua Su^1,2, †^, Jia-yan Huang^1,2^, Jun Liu^4^,*, Zhen-feng Chen^1,2^,* and Ke-guang Cheng^1,2^,*

**^1^**State Key Laboratory for the Chemistry and Molecular Engineering of Medicinal Resources, Guangxi Normal University, Guilin 541004, People’s Republic of China

**^2^**School of Chemistry and Pharmacy, Guangxi Normal University, Guilin 541004, People’s Republic of China

**^3^**Biochemistry and Pharmacology of Sport School, Guangxi Normal University, Guilin 541004, People’s Republic of China

**^4^**Jiangsu Key Laboratory of Drug Screening, China Pharmaceutical University, 24 Tongjia Xiang, Nanjing 210009, People’s Republic of China

**^†^** Wei-bin Mo and Chun-hua Su contributed equally to this work.

**Table of Contents**

Copies of NMR spectra of compound **4a**………………………………………...S1-S2

Copies of NMR spectra of compound **4b**………………………………………...S3-S4

Copy of NMR spectrum of compound **4c** ……………………………………… S5-S6

Copies of NMR spectra of compound **4d**………………………………………...S7-S8

Copy of NMR spectrum of compound **4e** ………………………………………....S9

^1^H NMR spectrum of **4a**


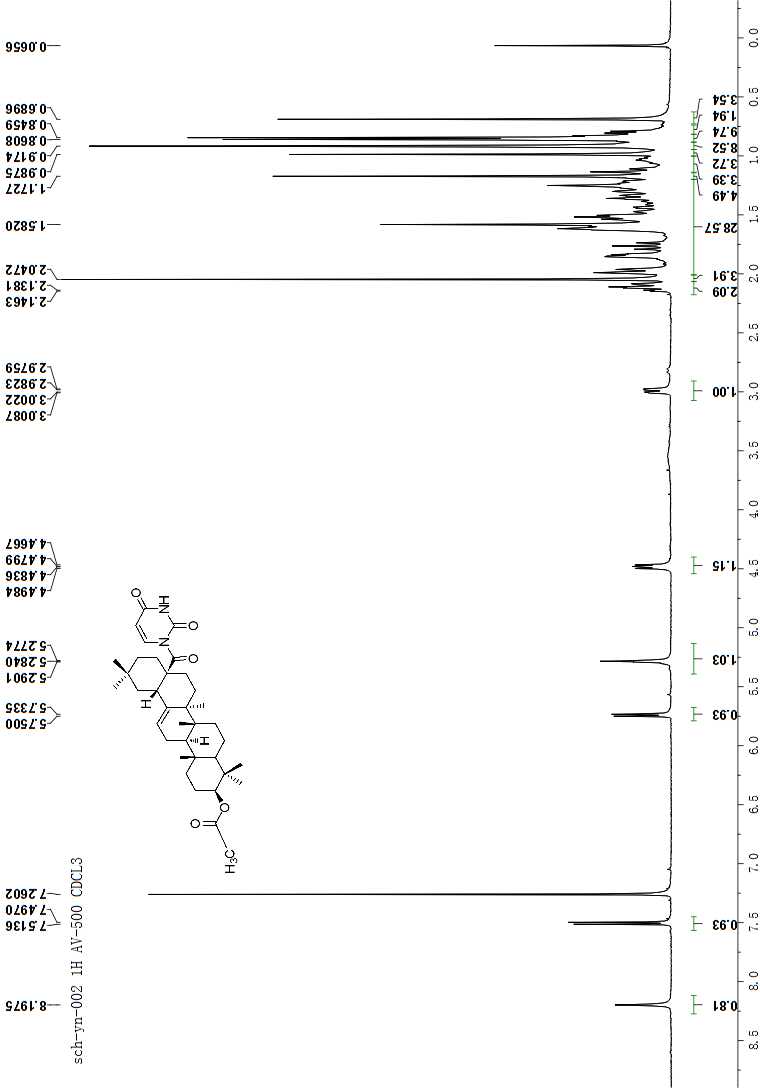


^13^C NMR spectrum of **4a**


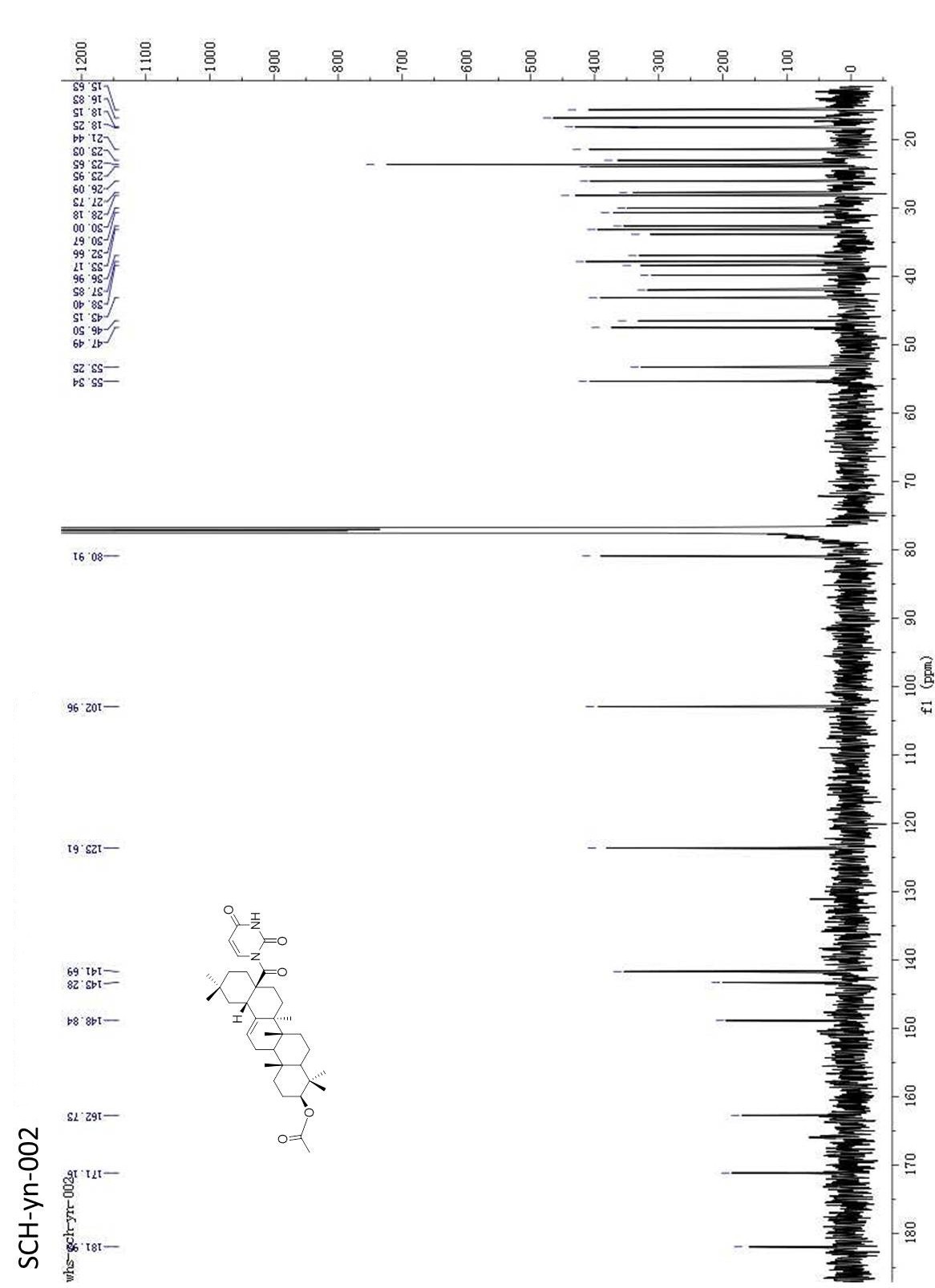


^1^H NMR spectrum of **4b**


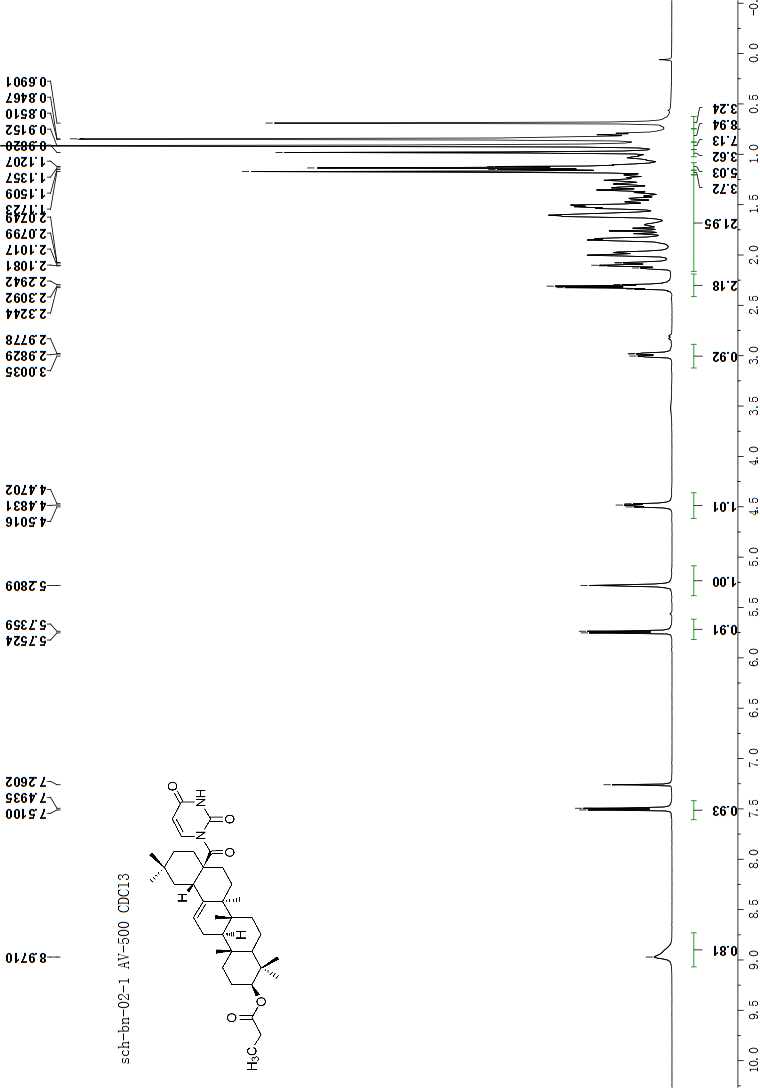


^13^C NMR spectrum of **4b**


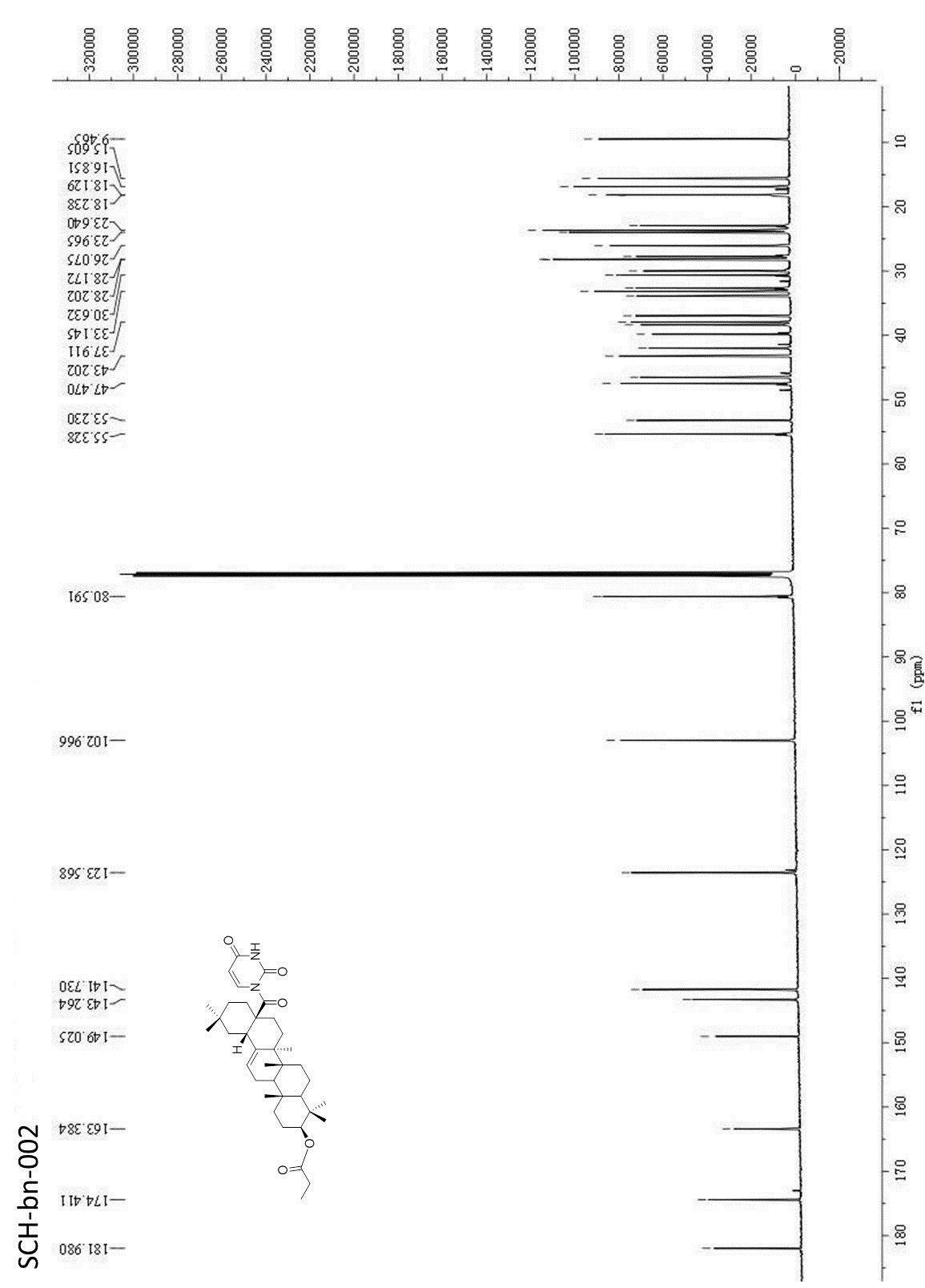


^1^H NMR spectrum of **4c**


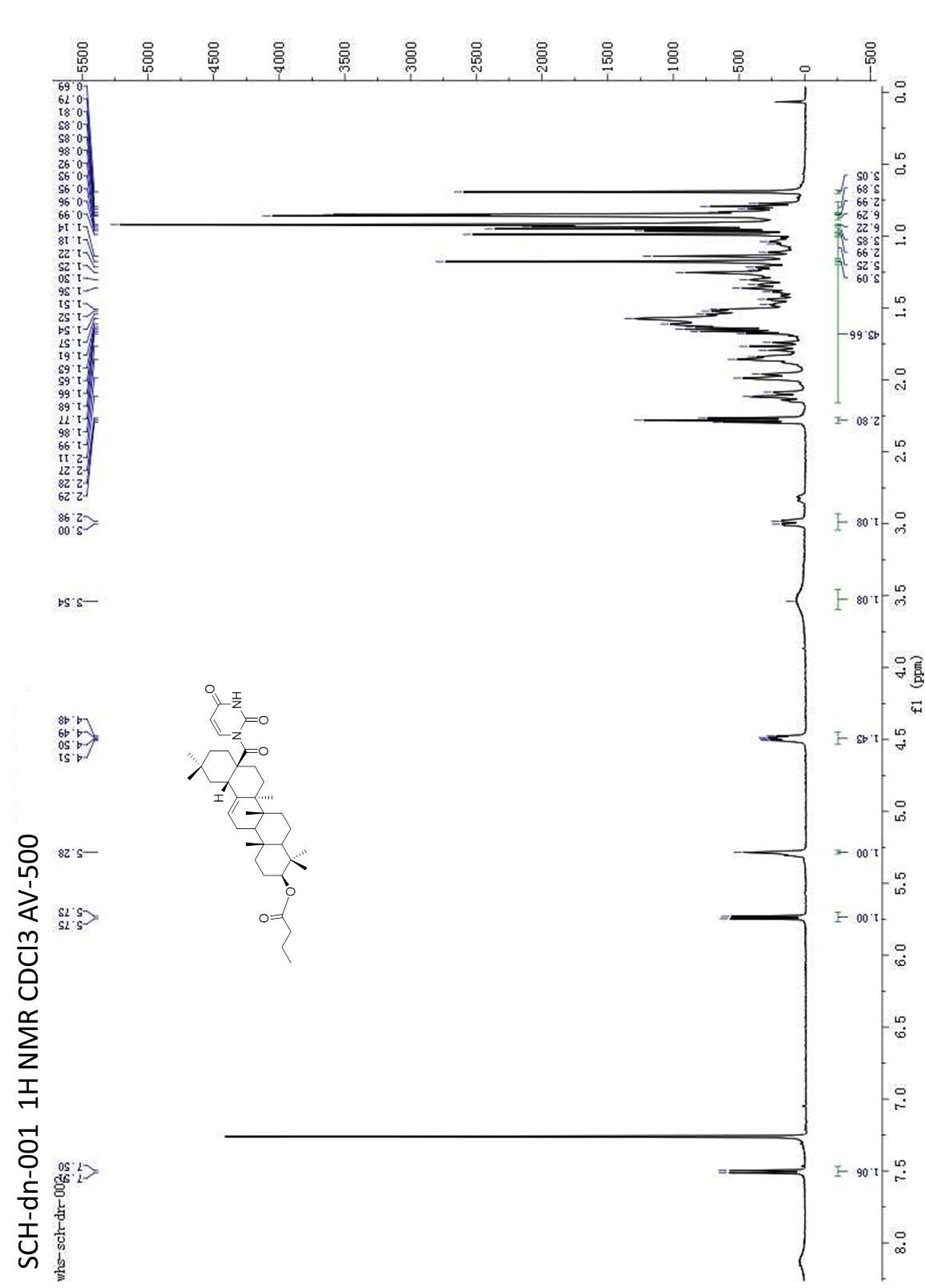


^13^C NMR spectrum of **4c**


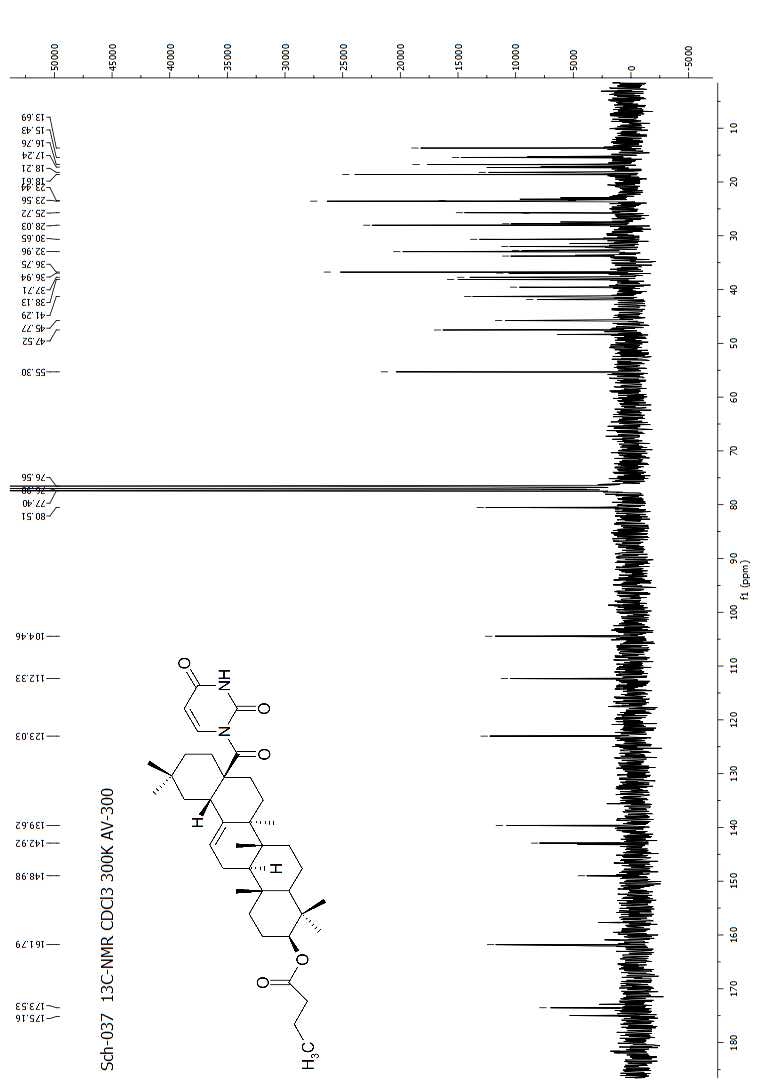


^1^H NMR spectrum of **4d**


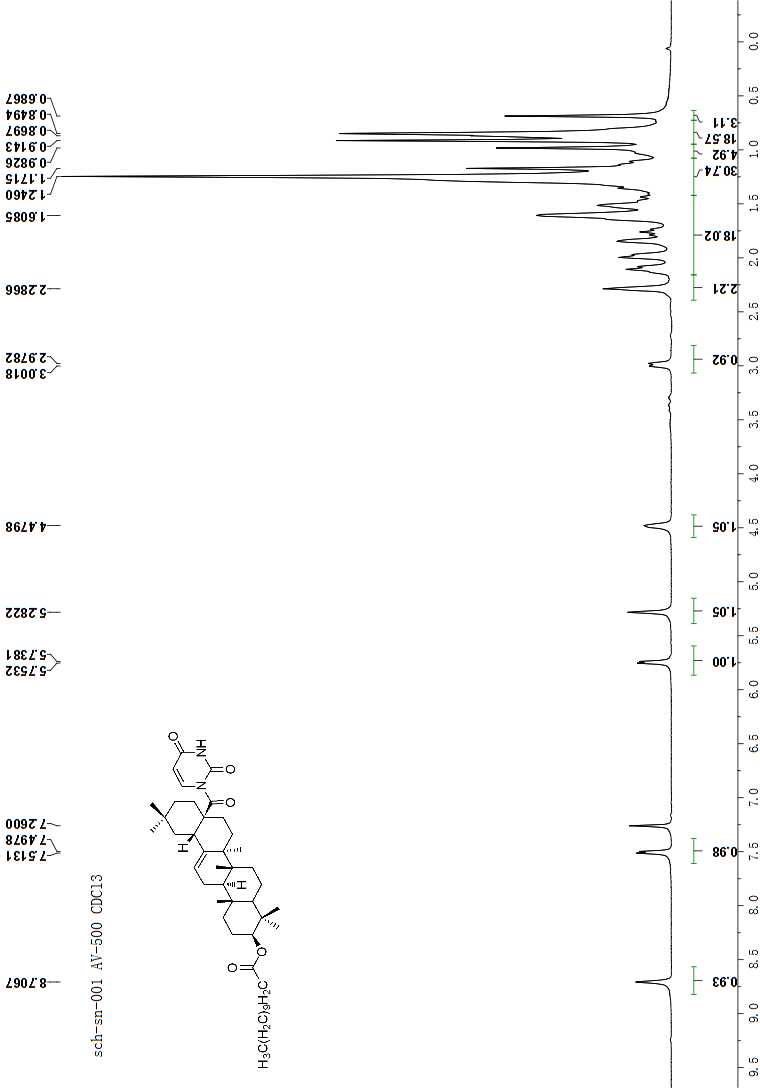


^13^C NMR spectrum of **4d**


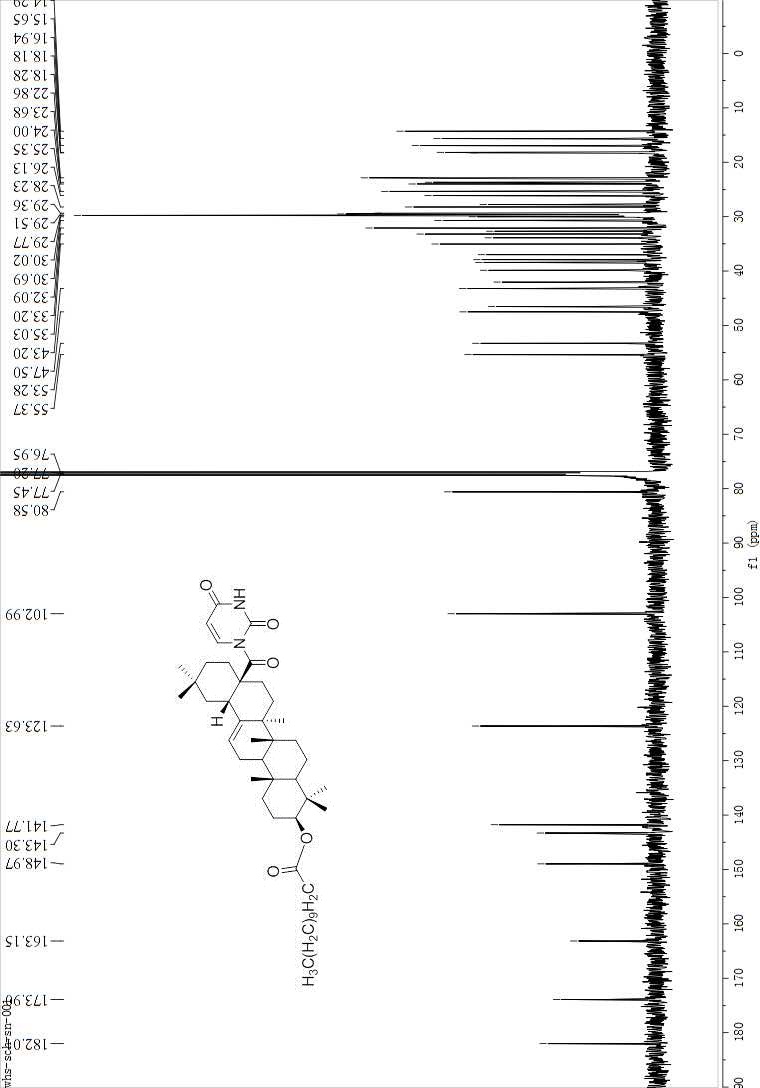


^1^H NMR spectrum of **4e**


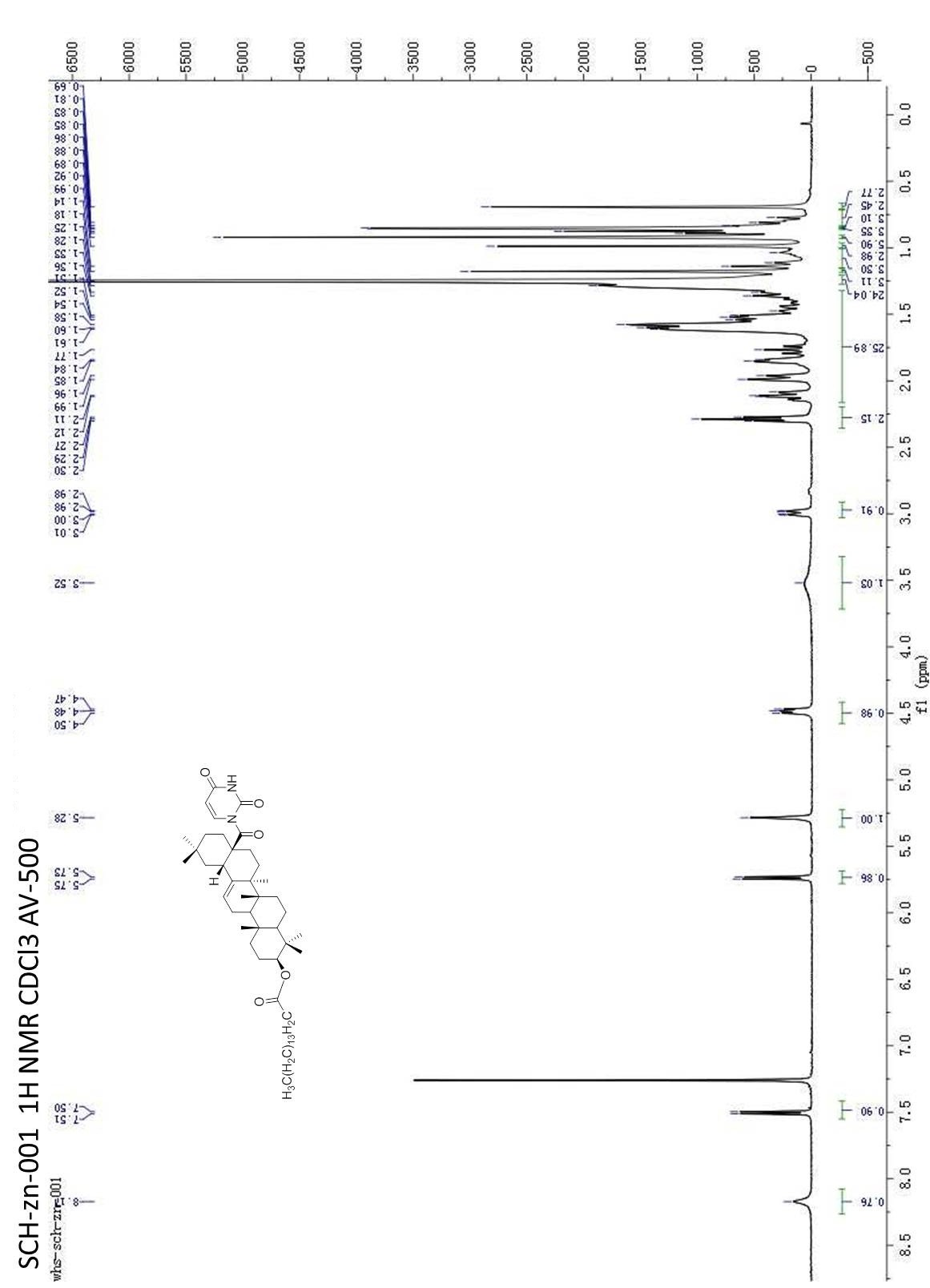

Supplement: Supplementary file 1 — Additional file 1. The copies of NMR spectra of compounds 4a–4e [file 13065_2016_217_MOESM1_ESM.doc]
